# Double sticker over knife to assess myotomy and mucosectomy length during Zenker’s peroral endoscopic myotomy for Zenker’s diverticulum

**DOI:** 10.1055/a-2443-6374

**Published:** 2024-11-08

**Authors:** Miguel Fraile-López, Carmen Ribes, Álvaro Terán, María Moris, Juan Carlos Rodríguez, María Jesús López Arias, Antonio Cuadrado Lavín

**Affiliations:** 1 Gastroenterology and Hepatology Department, Clinical and Translational Research in Digestive Diseases, Valdecilla Research Institute (IDIVAL), Marqués de Valdecilla University Hospital, Santander, Spain

Flexible endoscopic therapies are becoming increasing popular for the treatment of Zenker’s diverticulum. In particular, Zenker’s diverticulum peroral endoscopic myotomy (zPOEM) allows a selective and precise cut of the cricopharyngeal muscle under direct visualization, improving its effectiveness and safety 1 . A major concern regarding the length cut is the risk of perforation and/or difficulties in the incisional closure if the cut is extended beyond the fundus, or the risk of recurrence of dysphagia if the procedure is incomplete and/or there is an excess of mucosal flap remaining.

As previously described for myotomy length in achalasia 2 , the “double sticker over knife” technique permits a precise measurement of Zenker’s diverticulum depth and individualization of the exact length of myotomy and complementary cut of the mucosal flap required. We used the recently described “noninjection nontunnel technique” (Ni-zPOEM) using a scissor-type knife (Clutch-Cutter DP2618DT; Fujifilm Medical, Tokyo, Japan), which does not require submucosal injection or tunneling 3 .

First, the scope is advanced close to the diverticular septum, and a sticker is placed on the sheath of the knife externally, aligned with the opening of the working channel ( Fig. 1 **a** ). With the scope held in this position, the knife is advanced further through the working channel until the tip reaches the fundus of the Zenker’s diverticulum. A second sticker is then placed on the knife sheath externally, again aligning it with the opening of the working channel ( Fig. 1 **b** ). These two reference points allow us to precisely measure the Zenker’s diverticulum depth ( Fig. 1 **c** ). Ni-zPOEM continues with mucosal incision and selective myotomy. The scope is then placed again at the septum and the tip of the knife is advanced through the tunnel until the tip reaches the muscle. Here, the second sticker should align with the working channel as it did initially ( Fig. 1 **d** ).

**Fig. 1:**
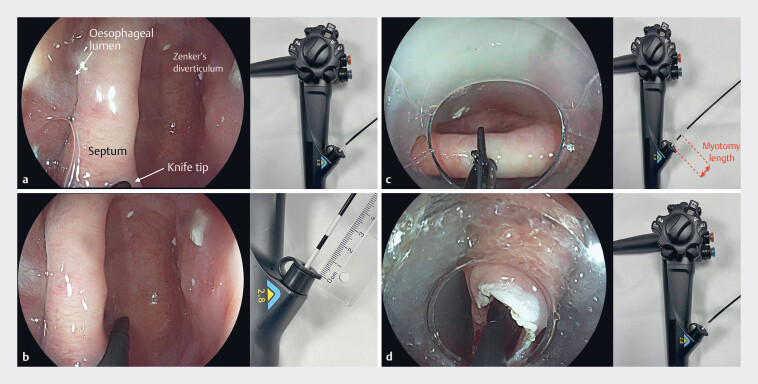
Double sticker over knife technique. **a** The tip of the knife is placed in contact with the septum. A sticker is placed on the knife sheath, aligned with the opening of the working channel. **b** Without moving the scope, the knife is advanced through the working channel until the tip of the knife reaches the fundus of the Zenker’s diverticulum. A second sticker is placed over the knife sheath. The distance between the two stickers is the desired myotomy length. **c, d** Mucosal incision and tunneling myotomy is performed until the second sticker reaches the opening of the working channel.

Our experience consists of 20 cases with a mean Zenker’s diverticulum size of 26.7 (SD 12.7) mm. Technical success was achieved in all 20 cases (100%) with a mean procedure time of 32.4 (SD 12.8) minutes. Clinical success at 6 weeks’ follow-up was 100% ( Video 1 ).

Use of the “double sticker over knife” technique to assess myotomy and mucosectomy length during Zenker’s diverticulum peroral endoscopic myotomy.Video 1

Endoscopy_UCTN_Code_TTT_1AO_2AP

## Notes

Endoscopy E-Videos https://eref.thieme.de/e-videos
*E-Videos* is an open access online section of the journal *Endoscopy* , reporting on interesting cases and new techniques in gastroenterological endoscopy. All papers include a high-quality video and are published with a Creative Commons CC-BY license. Endoscopy E-Videos qualify for HINARI discounts and waivers and eligibility is automatically checked during the submission process. We grant 100% waivers to articles whose corresponding authors are based in Group A countries and 50% waivers to those who are based in Group B countries as classified by Research4Life (see: https://www.research4life.org/access/eligibility/ ). This section has its own submission website at https://mc.manuscriptcentral.com/e-videos .

## References

[1] Steinway S Zhang L Amundson J Long-term outcomes of Zenker’s peroral endoscopic myotomy (Z-POEM) for treatment of Zenker’s diverticulum Endosc Int Open 2023 11 E607 E612 10.1055/a-2067-9105 37397859 PMC10310448

[2] Uchima H Colán-Hernández J Aguilar A A simple method to determine the proper length of the gastric myotomy during peroral endoscopic myotomy for achalasia Endoscopy 2022 54 E85 E87 33723848 10.1055/a-1388-6444

[3] Gorrepati VS Yang D Draganov PV Novel non-injection non-tunnel technique for peroral endoscopic myotomy of Zenker’s diverticulum VideoGIE 2023 8 181 182 10.1016/j.vgie.2023.01.001 37197163 PMC10183661

